# Improvement of Quality of Life with Implant-Supported Mandibular Overdentures and the Effect of Implant Type and Surgical Procedure on Bone and Soft Tissue Stability: A Three-Year Prospective Split-Mouth Trial

**DOI:** 10.3390/jcm8060773

**Published:** 2019-05-31

**Authors:** Ron Doornewaard, Maarten Glibert, Carine Matthys, Stijn Vervaeke, Ewald Bronkhorst, Hugo de Bruyn

**Affiliations:** 1 Department Periodontology & Oral Implantology, Dental School, Faculty Medicine and Health Sciences, Ghent University, De Pintelaan 185, 9000 Ghent, Belgium; maarten.glibert@ugent.be (M.G.); carine.matthys@ugent.be (C.M.); stijn.vervaeke@ugent.be (S.V.); 2 Section Implantology & Periodontology, Department of Dentistry, Radboudumc, Philips van Leydenlaan 25, 6525 EX Nijmegen, The Netherlands; ewald.bronkhorst@radboudumc.nl

**Keywords:** bone loss, dental implant, overdenture, implant survival, peri-implantitis, implant surface, soft tissue, split-mouth design, oral health-related quality of life, patient-reported outcome measures

## Abstract

In fully edentulous patients, the support of a lower dental prosthesis by two implants could improve the chewing ability, retention, and stability of the prosthesis. Despite high success rates of dental implants, complications, such as peri-implantitis, do occur. The latter is a consequence of crestal bone loss and might be related to the implant surface and peri-implant soft tissue thickness. The aim of this paper is to describe the effect of implant surface roughness and soft tissue thickness on crestal bone remodeling, peri-implant health, and patient-centered outcomes. The mandibular overdenture supported by two implants is used as a split-mouth model to scrutinize these aims. The first study compared implants placed equicrestal to implants placed biologically (i.e., dependent on site-specific soft tissue thickness). The second clinical trial compared implants with a minimally to a moderately rough implant neck. Both studies reported an improvement in oral health-related quality of life and a stable peri-implant health after three years follow-up. Only equicrestal implant placement yielded significantly higher implant surface exposure, due to the establishment of the biologic width. Within the limitations of this study, it can be concluded that an implant supported mandibular overdenture significantly improves the quality of life, with limited biologic complications and high survival rates of the implants.

## 1. Introduction

Edentulousness is widely spread worldwide. According to the WHO the prevalence in the elderly population is 26% in the USA and between 15% and 78% in European countries. Among the edentulous population, a strong negative impact of poor oral conditions on daily life has been described. Edentulism could lead to diet changes where food rich in saturated fats and cholesterol are preferred. Besides diet changes, edentulousness is an independent risk factor for weight loss and could lead to social handicaps related to communication [1].

The support of a dental prosthesis by two implants could improve the chewing ability, retention, and stability of the prosthesis, which could lead to higher satisfaction and health-related quality of life. Dental implants have been used since the early sixties to replace missing teeth by fixed or removable prostheses. Nowadays, this yields a predictable treatment outcome with success over 95% after 10 years of function [2].

To measure the improvement in health-related quality of life, the Oral Health Impact Profile (OHIP) is a widely used tool to assess currently applied dental procedures. It has also been used for evaluating the quality of life in more invasive surgical interventions in oral surgery [3]. The tool consists of a questionnaire to measure the impact of medical care on functional and social wellbeing [4]. Allen and McMillan reported significant improvement in satisfaction and health-related quality of life for patients who received implant-retained prostheses compared to those who received conventional dentures [5]. A panel of experts published a consensus statement where they described overwhelming evidence for a 2-implant supported overdenture as the first choice of treatment for the edentulous mandible instead of a conventional denture [6].

A recent review focusing on the Patient-Reported Outcome Measures (PROMs) showed compelling evidence to support that the fully edentulous patients experience higher satisfaction with an implant-supported overdenture in the mandible compared to a conventional denture [7]. These findings were confirmed by several other recent systematic reviews and meta-analyses [8,9,10].

De Bruyn and co-workers also concluded that patient satisfaction is highly individual and satisfaction with an implant-supported overdenture is never guaranteed. Hence, the decision to propose an implant-supported overdenture should be based on proper individual assessment [7]. 

Despite the improvement of the patient’s quality of life and high survival and success rates of dental implants in patients with overdentures, dental implants are not free of complications. The most common complications following implant therapy are peri-implant mucositis (bleeding on probing and inflammation of the peri-implant soft tissues), and peri-implantitis (clinical and radiographic bone loss with or without suppuration). To detect inflammatory changes around the implant, several biologic parameters (plaque, bleeding, and suppuration) must be monitored during the patient’s follow-up visits [11].

According to the latest consensus report of the “World Workshop on the Classification of Periodontal and Peri-implant Diseases and Conditions”, the main clinical characteristic of peri-implant mucositis is bleeding on gently probing [12]. Erythema, swelling, and/or suppuration may also be present [13]. There is strong evidence from animal and human experimental studies that plaque is the etiological factor for peri-implant mucositis [11,14,15,16,17,18]. Peri-implantitis is described as a plaque-associated pathologic condition occurring in tissues around dental implants, characterized by inflammation in the peri-implant mucosa and subsequent progressive loss of supporting bone. Peri-implantitis sites exhibit clinical signs of inflammation, bleeding on probing, and/or suppuration, increased probing depths and/or recession of the mucosal margin in addition to radiographic bone loss [19]. Peri-implantitis is a consequence of crestal bone loss. Two recent consensus meetings highlighted the influence of implant material, shape and surface characteristics on the occurrence and progression of peri-implantitis. However, evidence for these suggestions is weak and future long-term studies are necessary to analyze these potential risk factors [20,21]. Beside these implant factors also other important factors like surgical, prosthetic, patient-related factors and foreign body reactions may contribute to crestal bone loss [21].

The composition and the topography of the implant surface have been a matter of debate during the last decades. Both composition and topography have their influence on implant surface roughness. The implant surface roughness is expressed in a Sa value. This three-dimensional value expresses an absolute difference in the height of each point compared to the arithmetical mean of the surface [22]. In the early years of implant dentistry two types of implant surfaces were used, the machined/turned surface (Sa = 0.5–1 μm) and the microporous titanium plasma-sprayed surface (Sa > 2 μm). The first one is smooth and the latter could be described as a rough implant surface. Surface modification was done to enlarge the surface, resulting in a greater bone-to-implant contact area. Implant surface modifications were done by sandblasting, acid-etching, anodic oxidation or hydroxyapatite coating. These techniques resulted in a moderately rough implant surface (Sa = 1–2 μm), which is nowadays the most used surface roughness. Beside the higher bone-to-implant contact [23], a lower clinical failure rate [24] and a higher removal torque was observed compared to the smooth implant surfaces [25]. Hence, the surface modification made it possible to load the implant earlier or even immediately after the surgery. The resulting surface enlargement allowed shorter implants to be used, without jeopardizing the prognosis and with a reduced necessity for bone grafting procedures [2]. Beside the aforementioned benefits, related to faster integration, rough implant systems have been linked to increased bacterial adhesion [26]. The applied model in the latter study does not always mimic the clinical reality. However, A Cochrane systematic review suggested limited evidence that smooth surfaces had a 20% reduced risk of being affected by peri-implantitis over a three-year period [27,28]. This finding led to the commercial production of hybrid dental implants, combining the best of both systems. Hybrid dental implants have a minimally rough coronal part to decrease biofilm formation in the soft tissue crevice and a moderately rough implant body to enhance bone healing and speed up the osseointegration. These hybrid surfaces combine the effect of both surface roughnesses in the same implant. A short-term study indicated that the moderately rough and smooth coronal part showed the same crestal bone remodeling in the initial healing phase [29]. However, long-term studies to describe clinical parameters and peri-implant health are not yet available.

Some patient-related factors, such as certain metabolic syndrome components, medical conditions and/or the use of medication are known to have an effect on implant treatment outcome. Systematic reviews reveal that hyperglycemia has an increased risk for peri-implantitis [30,31], although the risk for more implant failures is comparable with the one observed in healthy patients. [32]. There is inconsistent and controversial evidence about the association with cardiovascular diseases [31]. Another meta-analysis revealed that there was no difference in implant survival rate between patients with and without osteoporosis. However, increased peri-implant bone loss was observed [33]. The intake of bisphosphonates, related to the treatment of osteoporosis, was not associated with an increased implant failure rate [34]. On the other hand, the same systematic review revealed an increased risk for implant failure with the intake of certain selective serotonin reuptake inhibitors and proton pump inhibitors [34]. Patients that are periodontally compromised are at higher risk for implant failure and crestal bone loss when compared with periodontally healthy subjects [35].

Another patient factor related to the failure of integrated implants is smoking. De Bruyn and Collaert described in a large retrospective study significantly higher failure rates of dental implants in smokers compared to non-smokers, both before and after functional loading, especially in the maxilla [36]. These findings are in agreement with a large meta-analysis of 18 studies showing an odds-ratio of 2.17 for implant failures in smokers were compared to non-smokers [37]. Besides implant failure smokers are more prone to peri-implant bone loss [38,39].

Also, biologic variances between patients could influence crestal bone loss around dental implants. Especially, soft tissue dimensions could play an important role in bone remodeling. The effect of peri-implant mucosal tissue thickness on the crestal bone loss was described in an animal study suggesting a certain minimal width of peri-implant mucosa may be required, and that bone resorption may take place allowing a stable soft tissue attachment [40]. The latter was confirmed in a human clinical trial, when there was a soft tissue thickness of 2 mm or less, crestal bone loss up to 1.45 mm may occur [41].

More recently Vervaeke and co-workers concluded that the initial bone remodeling was affected by the thickness of the peri-implant soft tissue [42]. They suggested that bone loss directly after implant placement, due to crestal bone remodeling, precludes the biologic width re-establishment and can be controlled by adapting the vertical depth position of the implant in the bone in relation to the soft tissue thickness at the time of implant placement. Hence, in thin tissues, a deeper subcrestal position in the bone may prevent partial exposure of the crestal part of the implant. Although crestal bone remodeling is a given fact after implant placement, related to the surgical trauma from periosteal elevation, as well as the drilling procedure, it is from a preventive point of view important to have the bone covering the implant as much as possible. Initial crestal bone loss, resulting in the absence of bone contact, can predict a future bone loss in patients prior to the disease. Galindo-Moreno and co-workers concluded that 96% of implants with a marginal bone loss above 2 mm at 18 months had lost 0.44 mm or more at six months post loading [43]. A critical long-term study where implants were placed in the partially edentulous mandible, indicated that bone loss in patients with thin (<2 mm) and a thick mucosa (>2 mm) was identical, when the implants were installed subcrestally to anticipate on the biologic width re-establishment [44].

Another subject of debate is the predictive value of biologic parameters around dental implants. Bleeding on probing, suppuration, plaque formation and probing pocket depth are the most widely used clinical parameters to describe health and/or disease around dental implants. These biologic parameters are most of the times included in the definition of peri-implantitis. However, a largely critical review showed the absence of a correlation between bone loss and the biologic parameters mean probing pocket depth and mean bleeding on probing. The authors also reported inconsistency and incompleteness in reporting on these parameters in the literature, which could affect decision-making in clinical practice [45].

Hence, the aim of this paper is to describe, by means of two prospective clinical split-mouth cohort studies, the effect of implant surface roughness and surgical implant depth positioning on crestal bone remodeling, peri-implant health, and patient-centered outcomes. The mandibular overdenture supported by two dental implants is used as a split-mouth model to scrutinize these aims.

## 2. Experimental Section

### 2.1. Patient Population and Surgical/Prosthetic Procedures

This paper includes two prospective split-mouth studies. Both studies included edentulous patients in need of a two-implant supported overdenture in the lower jaw. The same inclusion and exclusion were used for both studies. Inclusion criteria include: (1) Total complete edentulism for at least four months and (2) presence of sufficient residual bone volume to install two implants of 3.5 to 4.0 mm diameter and 8 to 11 mm length. Patients were excluded if they were: (1) Younger than 21, (2) suffered from systemic diseases, (3) current smokers and (4) had general contraindications for oral surgery (full dose head and neck radiation, intravenous administrated bisphosphonates, and ongoing chemotherapy). All patients were treated at the Ghent University Hospital by the same surgeon between January 2013 and September 2014. Twenty-six patients (study 1) received two moderately rough dental implants (Astra Tech Osseospeed TX™, Dentsply implants, York, PA, USA). The control implant was installed equicrestally (group 1), according to the manufacturer’s guidelines with the rough implant surface completely surrounded by bone. The vertical position of the test implant (group 2) was adapted to the soft tissue thickness, allowing at least 3 mm space for biologic width establishment [42].

**Figure 1:**
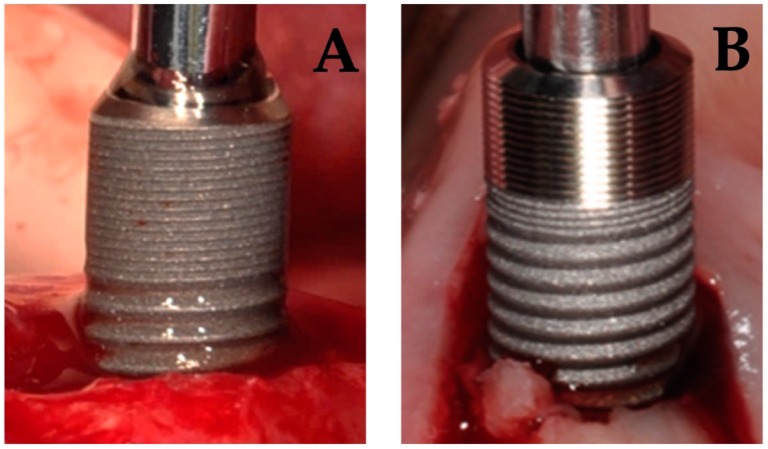
Placement of an implant with a moderately rough surface (**A**) and a hybrid implant with a minimally rough coronal neck (**B**).

Another 23 patients (study 2) received two dental implants with a difference in implant surface roughness of the coronal part of the implant (Figure 1). All 46 implants were biologically guided taking the soft tissue thickness into account whereby care was taken to ensure a 3 mm soft tissue seal in contact with the abutment. All patients received one moderately rough implant (group 3) (Sa = 1.3 μm) (DCC, Southern implants, Irene, South Africa) and one test implant (group 4). The latter was a hybrid dental implant with a minimally rough coronal neck of 3 mm (Sa = 0.9 μm) combined with a moderately rough body (Sa = 1.3 μm) (MSC, Southern implants, Irene, South Africa).

Although two different brands were used in both studies, all 98 implants installed in the 49 patients were identical at the level of the abutment-implant connection. Implants had the same integrated platform-shift with a smooth implant bevel, the same internal deep conical connection and a similar macro design of the micro-threads on the implant neck.

Implants were immediately restored if primary stability was achieved (insertion-torque > 25 Ncm). Implants were restored either with locator abutments (study 1) or definitive titanium abutments (Compact Conical Abutments; Southern Implants, Irene, South Africa) and a healing cap with a standard abutment height of 4 mm (study 2).

Before surgery, all patients received new removable dentures in the mandible and maxilla to achieve a correct occlusion, appropriate teeth position, and appropriate smile line. The removable dentures were adapted after surgery to connect with the implants by one experienced prosthodontist. The surgical and prosthetic procedures have been described previously by Vervaeke and co-workers [46] and Glibert and co-workers [29].

The clinical trial has been conducted in full accordance with the Helsinki Decleration (1975) as revised in 2000. All patients were thoroughly informed and signed written informed consent. The study protocol was approved by the ethical committee of the Ghent University Hospital.

### 2.2. Clinical and Radiographic Examination

Follow-up visits were planned at 1 week, 1, 3, 6, 12, 24, and 36 months after surgery. After soft tissue healing was fully established, three months after surgery, peri-implant health was monitored and probing pocket depths, bleeding on probing and plaque scores were assessed on four implants sites: Midmesial, middistal, midbuccal, and midlingual. The bleeding- and plaque scores were measured on a dichotomous scale (0 = absence of bleeding on probing/absence of plaque; 1 = bleeding on probing/plaque). From the site level scores both for bleeding and plaque mean scores on implant level were calculated.

Digital peri-apical radiographs were taken at baseline (implant placement), at 3, 6, 12, 24, and 36 months using a guiding system in order to obtain the X-rays perpendicular to the film. The radiographic measurements were calibrated using the length of the implant, the distance between the threads or the diameter of the implant. Bone levels were determined as the distance from a reference point, which corresponds with the lower edge of the smooth implant bevel at the implant-abutment interface, to the most crestal bone-to-implant contact point. The baseline bone-to-implant contact levels are assessed from the implant-abutment interface. The baseline from the four experimental groups was logically comparable. Bone loss was determined by the difference of the bone level directly after implant placement and the bone level at the follow-up visit.

If necessary, calculus and plaque were removed and oral hygiene was reinforced during follow-up visits. Instructions with a (electric) toothbrush and interdental brushes were given based on the need, preferences and dexterity or motoric skills of the patient.

To measure the change in oral health-related quality of life the Oral Health Impact Profile-14 questionnaire (OHIP-14) is assessed before surgery, 3, and 12 months after connection of the prosthesis with the implants (Table 1). The questionnaire is based on 14 questions capturing seven domains: Functional limitation, physical pain, psychological discomfort, physical disability, psychological disability, social disability, and handicap. Of these seven domains, two questions need to be answered on a Likert scale. Score 4 is indicating a highly negative answer to the question and 0 means that there is no discomfort at all. The total score of the 14 questions can balance between 56 (maximally negative) to 0 (maximally positive).

### 2.3. Statistics

Outcomes are reported with descriptive statistics (mean, SD, median, range, and 95% CI) and boxplots. All analyses concern pair-wise comparisons within patients. For continuous variables paired *t*-tests were applied, for dichotomous variables the McNemar test was used. The 95% confidence intervals are given to show the precision of an estimate of a certain effect.

The sample size for both studies was calculated using SAS Power and Sample size calculator for related samples based on an effect size of 1 mm and a standard deviation of 0.60, with the level of significance set at 0.05 and β = 0.80. The effect estimation was based on findings Vervaeke et al., 2014 [42].

For the OHIP-14 outcome, the impact of the change was assessed by calculating the “effect size” with the following formula:

((mean-OHIP before surgery) − (mean-OHIP three months after connection))/SD before surgery

As proposed by Cohen 1977 an “effect size” of 0.2 could be interpreted as a small change, 0.6 as a moderate change and > 0.8 as a large change.

## 3. Results

### 3.1. Study Population

A sample size of 14 patients for each study was calculated. Hence, minimums of 20 patients (= 40 implants) were consequently included to anticipate future dropouts.

**Figure 2:**
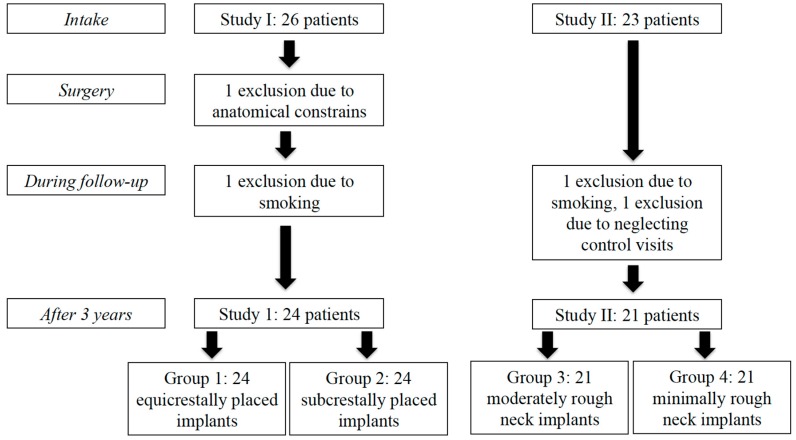
Flowchart of both study populations.

Twenty-six patients in study I were initially treated with one equicrestally (group 1) and one subcrestally (group 2) placed implant. In study II, 23 patients were initially treated with one implant with a moderately rough implant neck (group 3) and one implant with a minimally rough implant neck (group 4). In total four experimental treatment groups were assessed. After a follow-up of at least three years, one patient was excluded, due to anatomical constraints requiring deviation of the surgical protocol. Two patients were excluded after starting smoking and one did not respond to the follow-up invitation. Hence, 45 patients with two implants each were available after a follow-up of three years and none of the implants had failed (survival 100%). A flowchart of the patients’ distribution is shown in Figure 2. The study population consisted of 24 men and 21 women with a mean age at implant placement of 64 years (SD = 9.25, range = 43–85).

### 3.2. Mean Bone Level Difference

**Figure 3:**
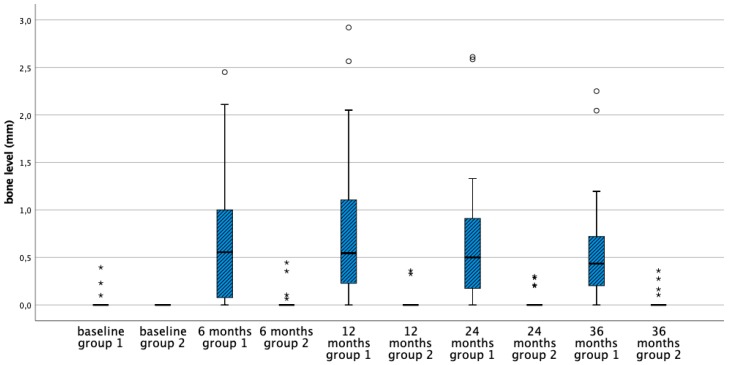
Boxplots representing the bone level at subsequent time points for the equicrestally (group 1) and subcrestally placed implants (group 2). * Outliers (≥3 × IQR above third quartile), ° suspected outliers (between 1.5 × IQR and 3 × IQR above third quartile).

**Figure 4:**
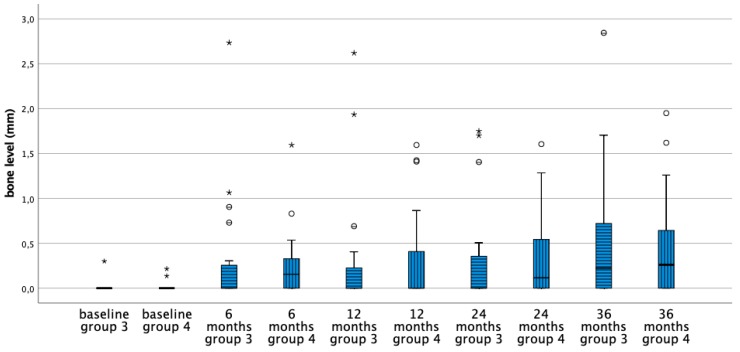
Boxplots representing the bone level at subsequent time points for the implants with a moderately rough neck (group 3) and minimally rough neck (group 4). * Outliers (≥3 × IQR above third quartile), ° suspected outliers (between 1.5 × IQR and 3 × IQR above third quartile).

**Figure 5:**
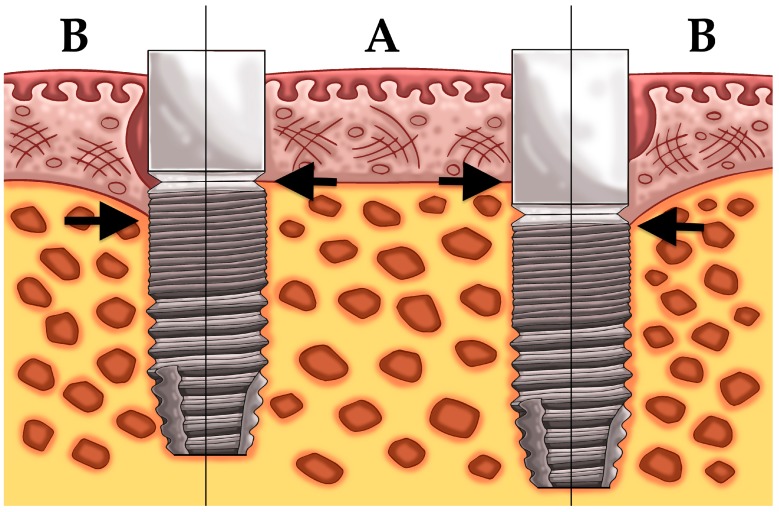
Schematic illustration of study 1, left equicrestally placed implant (group 1) and right subcrestally placed implant (group 2); showing the bone level at baseline (A) and bone level after bone remodeling (B).

**Figure 6:**
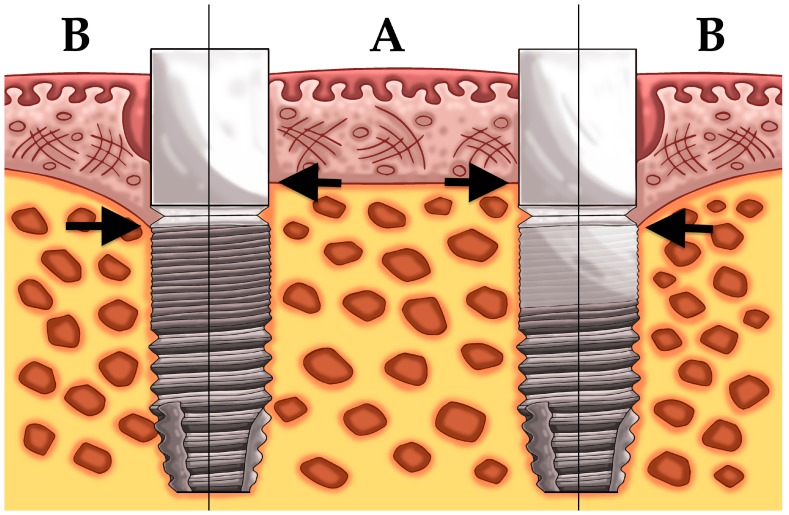
Schematic illustration of study 2, left implant with a moderately rough neck (group 3) and right implant with a minimally rough neck (group 4); showing the bone level at baseline (A) and bone level after bone remodeling (B).

Table 2 shows the mean bone level and the corresponding changes of the four treatment groups at baseline and after 6, 12, 24, and 36 months. Initially, the bone level of the implants in the four groups is comparable and basically located at the implant crest. In the first six months bone remodeling was 0.7 mm for equicrestally placed implants and ranging from 0–0.3 mm in the other three subcrestally placed groups. Over time no further statistically significant bone level changes occurred in all groups (Figure 3, Figure 4, Figure 5 and Figure 6). Figure 5 and Figure 6 gives a schematic view of the bone remodeling over time, with the visible implant surface exposure in the equicrestally placed implant group (group 1).

Between groups the subcrestally placed implants of group 2 lost no bone at all. Groups 3 and 4 showed comparable bone remodeling. Hence, implant surface roughness did not affect initial nor long-term bone remodeling (Figure 4 and Figure 6).

### 3.3. Biologic Parameters

On implant level only a statistically significant difference could be measured for the plaque score at 24 months (*p* = 0.042), with significantly less plaque for the equicrestally placed compared with subcrestally placed implants. However, at all other time points the plaque–and bleeding scores were not statistically significantly different, indicative of peri-implant health (Table 3).

For the probing pocket depth at implant level only at 24 months a statistically significant difference between equicrestally placed compared to subcrestally placed implants could be observed (Table 4). After three years all groups are comparable indicative of peri-implant health.

### 3.4. Oral Health-Related Quality of Life

**Figure 7:**
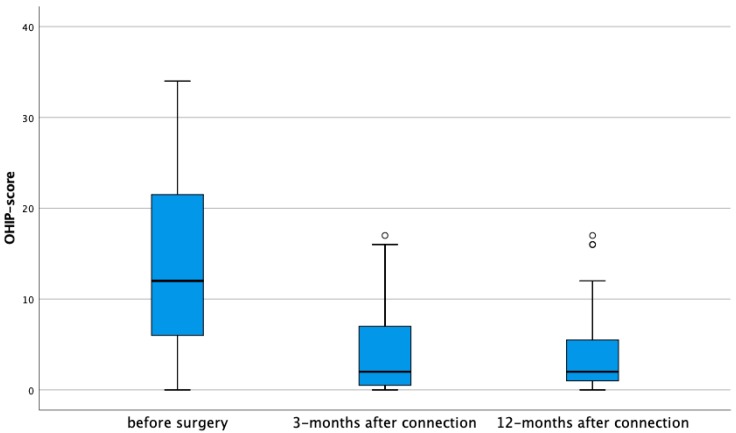
Boxplots representing the Oral Health Impact Profile-14 (OHIP-14) total score before surgery, 3 and 12 months after connection of the prosthesis with the implants. A score of 56 represents a maximal negative appreciation. ° Suspected outliers (between 1.5 × IQR and 3 × IQR above third quartile).

Based on 45 edentulous patients, receiving an implant-supported overdenture, the OHIP-14 index reduced from 13.37/56 (SD 9.97) at baseline to 4.42/56 (SD 4.94) after three months of functional loading. This result in a large effect size of 0.90, suggesting a strong improvement in oral health related quality of life. Between 3 and 12 months, no further changes were observed, resulting in small effect size (0.04), indicative of a very stable result over time (Figure 7). The reduction was statistically significant for all seven domains after three months (Table 5). For functional limitation, physical disability and handicap the effect size was moderate. For the other four domains, a large effect size was observed and most expressed for physical pain with an effect size of 1.04. The latter is logically given the fact that improved denture retention results in less mucosal irritation and consequently fewer complaints related to pain suffering.

## 4. Discussion

The current paper focuses on implant treatment outcome in patients, which were completely edentulous in both jaws. Retention of the lower denture is a typical problem in this category of patients, especially in the mandible as compared to the maxillary denture. The denture in the mandible is less retentive because of a smaller crestal bone support, a more expressed degree of bone resorption, and unfavorable distribution of occluding forces, as well as additional pressure of the tongue yielding dislocating forces. Often this results in functional discomfort and pain, the latter because of the absence of keratinized mucosa. In the maxilla, the denture is supported on the crest and on the hard structure of the palate, which is covered by keratinized tissue. A vacuum present during mastication, between the palatal coverage of the denture and the underlying tissues, improves the retention. Consequently, fully edentulous patients have more complaints with mandibular dentures and an overdenture retained on two implants has therefore been suggested as of minimal care in order to provide functional comfort [6]. Implant treatment in denture wearing patients can be used for split mouth studies as was the case in the two clinical studies presented in the present paper. The focus was on implant type and surgical procedure, defined as implant survival, crestal bone loss and biologic peri-implant health. The latter is an important aspect because peri-implant diseases may jeopardize treatment outcome in the long run and are often related to aesthetic appreciation. Additionally, the patient-centered outcome was assessed by using a validated Oral Health Related Quality of Life questionnaire.

After three years of follow-up, no implant failures could be recorded in the present study and all remaining patients remained fully functional. This 100% implant survival is in line with current literature on implant overdenture therapy [47].

Initial bone remodeling is a healing phenomenon related to the surgical procedure mainly the exposure of bone and periosteum during implant placement, as well as the depth placement in the bone. Given the fact that implant survival with currently available dental implant systems is successful and quite predictable, the research focuses on implant success. Implant treatment is considered a success when high implant survival is combined with bone stability over time, because the latter reflects the health of the peri-implant tissues. Indeed, worldwide consensus defined that peri implantitis, a disease condition of the implant resulting in pocket formation between the implant and soft tissue, is always preceded by the bone loss [12]. Additionally, soft tissue health also affects the aesthetic outcome, especially in the partially edentulous patient. Although aesthetics was not the key issue in the present paper, the study conditions tested may provide clinical guidelines that do affect aesthetics, as well as peri-implant health outcomes.

In the present paper, minimal initial bone remodeling ranging from 0–0.7 mm was assessed. After the physiological initial bone remodeling, no further bone loss could be observed up to three years of function. The effect of soft tissue thickness and implant surface roughness on the crestal bone loss was evaluated. The applied split-mouth study design corrects for inter-individual variability from the estimates of the treatment effect [48]. The results showed that the initial bone remodeling was affected by the originally present soft tissue thickness, but not by the implant surface roughness. After implant installation, a minimum of 3 mm soft tissue dimensions seems to be necessary for the re-establishment of the so-called “biologic width”, indicative of the importance of the biologically guided implant placement. These findings are in accordance with an earlier published systematic review, including meta-analysis. There it is stated that implants placed with an initially thicker peri-implant soft tissue have less radiographic marginal bone loss in the short term [49]. Additionally, an increased early bone remodeling leads to implant surface exposure in patients with thin soft tissues, which increases the risk of on-going bone loss as shown by Vervaeke and colleagues in a nine year follow-up. A greater implant surface exposure increases the bacterial colonization of the implant surface, which could enlarge the chance to induce peri-implantitis [50]. From a clinical point of view, it is highly suggested that the surgeon adapts the surgical position of the implant in relation to the available pre-operative soft-tissue thickness.

It is generally accepted that osseointegration of moderately rough implants is enhanced as compared to minimally rough implants. This resulted in faster treatment protocols and reduced early failures. More recently, it was suggested that a minimally rough implant surface yields less crestal bone loss and less peri-implantitis on the long-term. A recent systematic review, including studies up to 10 years, reported on the survival rate and marginal bone loss of implants with different surface roughness. Implant survival was higher for moderately rough surfaces, but minimally rough surfaces showed the least marginal bone loss [51]. This outcome is in contrast to the outcome presented in another systematic review with meta-analysis. The latter evaluated the influence of the implant collar surface on marginal bone loss and revealed less bone loss for the rougher implant systems. However, 10 out of the 12 included studies showed results with less than five years of function. The only study with 10 years of follow-up showed less bone loss for the implants with a smooth collar compared to the implants with a rough collar. Yet, the authors stated that the results of their systematic review needed to be interpreted cautiously, due to several confounding factors [52]. Another systematic review with meta-analysis, which included only studies with at least, a five-year follow-up showed significantly less bone loss around smooth implant surfaces compared to moderately rough and rough implant surfaces [38]. Recently Donati and co-workers published the results of a 20-year follow-up RCT to evaluate the effect of a modified implant surface. In 51 patients at least one implant with a minimally rough surface and one with a modified surface was installed. The difference in mean bone level change between the two implant-systems was not statistically significant, and the moderate increase of implant surface roughness has no beneficial effect on long-term preservation of the peri-implant marginal bone level. A more detailed analysis of the paper revealed, however, that none of the 32 evaluated smooth implants showed more than 3 mm bone loss, whereas 3 out of the 32 modified implants showed bone loss between 3 and 6 mm. Only two smooth surface implants were diagnosed with peri-implantitis compared with five implants with a modified surface [53]. 

The findings of our paper are in accordance with the paper of Donati and co-workers, concluding that the surface roughness of the implant neck has no effect on bone level up to three years. The hybrid implant system used in our study combines the benefits of faster osseointegration, due to the moderately rough implant body, and the minimally rough surface around the implant neck suggests it is less prone to develop peri-implantitis [54]. Additionally, several studies conclude the beneficial effect of a smoother surface with a lower incidence of peri-implantitis and less bone loss on the long term. A further long-term follow-up of the current study population will elucidate the latter.

Besides implant survival and bone level stability, also peri-implant health is considered a perquisite for treatment success. Peri-implant health is defined on two levels. Plaque accumulation yields minor inflammation of the soft tissue surrounding the implant- restorative interface, coined as mucositis. It is diagnosed with bleeding of the tissues after probing the crevice between implant and mucosa. In a recent consensus report, the diagnosis of peri-implantitis has been redefined as a combination of probing pocket depths of at least 6 mm in combination with bleeding on probing or a bone level of at least 3 mm apical of the most coronal portion of the intraosseous part of the implant [12]. In our study, no patients showed ongoing bone-loss in combination with bleeding and increasing probing pocket depths. Hence, the incidence of peri-implantitis was 0.0%.

The absence of peri-implantitis was found despite a high plaque level. This could be explained by the elderly, fully edentulous patient population. De Waal and colleagues revealed that edentulous patients restored with implants showed more plaque compared to partially edentulous patients restored with implants. However, the plaque in the fully edentulous patients harbours a potentially less pathogenic peri-implant micro-flora [55,56].

Another explanation for the relatively high plaque scores could be the dexterity problems inducing imperfect cleaning abilities in elderly patients. On the other hand, plaque is screened at a given moment in time during the clinical inspection and this may be several hours after cleaning and not necessarily reflects the overall hygiene of the patient over time.

This is the reason why the bleeding index is considered more useful. It reflects the degree of inflammation as a result of the long-term plaque control and is less momentarily. The current study revealed that high plaque score did not result in high bleeding scores.

The support of a mandibular overdenture by two implants has a significant positive effect on the quality of life. The OHIP-14 score was calculated irrespective of the implant group because it is a patient-related outcome variable. On all the seven domains measured with the OHIP-14 questionnaire a statistically significant difference was measured, all in favor of the support of a mandible overdenture by two implants. These findings are in accordance with a clinical trial reporting a significant improvement in satisfaction and health-related quality of life when subjects who received two implants are compared with subjects requesting a new conventional denture. Besides the improvement in the quality of life, they reported that patients requesting implants reported that tooth loss and denture wearing problems had a much greater impact in their quality of life than patients seeking conventional dentures [5].

## 5. Conclusions

Within the limitations of this study, it can be concluded that an implant supported mandibular overdenture significantly improves the quality of life, with limited biologic complications and a high survival rate of the implants. All seven domains of the OHIP-14 questionnaire significantly reduced when the mandible overdenture is supported by two implants. No differences were observed in crestal bone remodeling between minimally rough and moderately rough implant surfaces. However, initial bone remodeling was affected by initial soft tissue thickness. Anticipating biologic width re-establishment by adapting the vertical position of the implant in relation to the available soft tissue thickness may avoid peri-implant bone loss. The biologic variance of the patient might be more important compared to the configuration of the implant surface. Long-term follow-up of the study is necessary to determine the influence of early implant surface exposure and implant surface roughness on crestal bone loss, biologic parameters, mechanical complication, and implant survival.

## Figures and Tables

**Table 1:** OHIP-14 questionnaire divided per domain.

**Domain 1: Functional Limitation**	
1	Have you had trouble pronouncing any words because of problems with your teeth, mouth, or denture?
2	Have you felt that your sense of taste has worsened because of problems with your teeth, mouth, or denture?
**Domain 2: Physical Pain**	
3	Have you had painful aching in your mouth?
4	Have you found it uncomfortable to eat any foods because of problems with your teeth, mouth, or denture?
**Domain 3: Psychological Discomfort**	
5	Have you been self-conscious because of your teeth, mouth, or denture?
6	Have you felt tense because of problems with your teeth, mouth, or denture?
**Domain 4: Physical Disability**	
7	Has been your diet been unsatisfactory because of problems with your teeth, mouth, or denture?
8	Have you interrupt meals because of problems with your teeth, mouth, or denture?
**Domain 5: Psychological Disability**	
9	Have you found it difficult to relax because of problems with your teeth, mouth or denture?
10	Have you been a bit embarrassed because of problems with your teeth, mouth, or denture?
**Domain 6: Social Disability**	
11	Have you been a bit irritable with other people because of problems with your teeth, mouth, or denture?
12	Have you had difficulty doing your usual jobs because of problems with your teeth, mouth, or denture?
**Domain 7: Handicap**	
13	Have you felt that life, in general, was less satisfying because of problems with your teeth, mouth, or denture?
14	Have you been totally unable to function because of problems with your teeth, mouth, or denture?

**Table 2:** Mean bone level for each study group and the bone level difference between respectively equicrestally versus subcrestally placed implants and implants with moderately rough versus minimally rough neck; *p* < 0.05 indicates a statistically significant difference (paired *t*-test).

Bone Level											
**Group 1: Equicrestal**					**Group 2: Subcrestal**				**Paired Difference**		
	**Mean (SD)**	**Median**	**Min**	**Max**	**Mean (SD)**	**Median**	**Min**	**Max**	**Mean dif**	**95% CI**	***p***
Baseline	0.03 (0.09)	0.00	0.00	0.40	0.00 (0.00)	0.00	0.00	0.00	0.030	(−0.009,0.070)	0.123
6 months	0.72 (0.74)	0.59	0.00	2.45	0.04 (0.11)	0.00	0.00	0.45	0.678	(0.360,0.996)	<0.001
12 months	0.78 (0.81)	0.54	0.00	2.92	0.03 (0.10)	0.00	0.00	0.36	0.746	(0.397,1.096)	<0.001
24 months	0.69 (0.70)	0.51	0.00	2.61	0.04 (0.10)	0.00	0.00	0.30	0.644	(0.337,0.951)	<0.001
36 months	0.59 (0.59)	0.44	0.00	2.25	0.04 (0.10)	0.00	0.00	0.36	0.549	(0.297,0.802)	<0.001
**Group 3: Moderately Rough Neck**					**Group 4: Minimally Rough Neck**				**Paired Difference**		
	**Mean (SD)**	**Median**	**Min**	**Max**	**Mean (SD)**	**Median**	**Min**	**Max**	**Mean dif**	**95% CI**	***p***
Baseline	0.01 (0.07)	0.00	0.00	0.30	0.02 (0.05)	0.00	0.00	0.22	−0.002	(−0.424,0.037)	0.902
6 months	0.33 (0.64)	0.00	0.00	2.74	0.27 (0.38)	0.18	0.00	1.60	0.064	(−0.118,0.245)	0.474
12 months	0.34 (0.68)	0.00	0.00	2.62	0.34 (0.53)	0.00	0.00	1.61	0.009	(−0.191,0.209)	0.926
24 months	0.36 (0.58)	0.00	0.00	1.75	0.37 (0.49)	0.23	0.00	1.60	−0.014	(−0.170,0.142)	0.853
36 months	0.51 (0.74)	0.22	0.00	2.84	0.45 (0.58)	0.26	0.00	1.95	0.066	(−0.114,0.246)	0.453

**Table 3:** Mean plaque and bleeding on probing on implant level at 6, 12, 24 and 36 month for each study group and mean difference between respectively equicrestally versus subcrestally placed implants and implants with moderately rough versus minimally rough neck; *p* < 0.05 indicates a statistically significant difference (paired *t*-test).

**Plaque**					
	**Group 1: Equicrestal**	**Group 2: Subcrestal**	**Paired Difference**		
	**Mean (SD)**	**Mean (SD)**	**Mean dif**	**95% CI**	***p***
6 months	0.44 (0.47)	0.52 (0.45)	−0.083	(−0.221,0.055)	0.224
12 months	0.45 (0.39)	0.56 (0.44)	−0.115	(−0.285,0.056)	0.178
24 months	0.42 (0.40)	0.51 (0.40)	−0.091	(−0.178,−0.003)	0.042
36 months	0.39 (0.43)	0.41 (0.42)	−0.022	(−0.148,0.104)	0.724
	**Group 3: Moderately Rough Neck**	**Group 4: Minimally Rough Neck**	**Paired Difference**		
	**Mean (SD)**	**Mean (SD)**	**Mean dif**	**95% CI**	***p***
6 months	0.38 (0.33)	0.40 (0.31)	−0.025	(−0.144,0.094)	0.666
12 months	0.37 (0.31)	0.35 (0.31)	0.017	(−0.136,0.169)	0.818
24 months	0.57 (0.36)	0.52 (0.36)	0.054	(−0.030,0.137)	0.189
36 months	0.39 (0.41)	0.43 (0.38)	−0.038	(−0.147,0.072)	0.481
**Bleeding on Probing**					
	**Group 1: Equicrestal**	**Group 2: Subcrestal**	**Paired Difference**		
	**Mean (SD)**	**Mean (SD)**	**Mean dif**	**95% CI**	***p***
6 months	0.15 (0.22)	0.15 (0.22)	0.000	(−0.093,0.0933)	1.000
12 months	0.19 (0.18)	0.19 (0.18)	0.000	(−0.125,0.125)	1.000
24 months	0.23 (0.30)	0.20 (0.28)	0.023	(−0.090,0.136)	0.680
36 months	0.30 (0.33)	0.23 (0.25)	0.076	(−0.048,0.200)	0.216
	**Group 3: Moderately Rough Neck**	**Group 4: Minimally Rough Neck**	**Paired Difference**		
	**Mean (SD)**	**Mean (SD)**	**Mean dif**	**95% CI**	***p***
6 months	0.24 (0.31)	0.23 (0.24)	0.013	(−0.110,0.135)	0.834
12 months	0.20 (0.32)	0.23 (0.24)	−0.033	(−0.189,0.122)	0.653
24 months	0.25 (0.29)	0.30 (0.37)	−0.054	(−0.243,0.136)	0.551
36 months	0.08 (0.14)	0.07 (0.12)	0.013	(−0.084,0.109)	0.789

**Table 4:** Mean probing pocket depth on implant level at 6, 12, 24 and 36 months for each study group and the mean difference between respectively equicrestally versus subcrestally placed implants and implants with a moderately rough versus minimally rough neck; *p* < 0.05 indicates a statistically significant difference (paired *t*-test).

**Probing Pocket Depth**									
	**Group 1: Equicrestal**			**Group 2: Subcrestal**			**Paired Difference**		
	**Mean (SD)**	**Min**	**Max**	**Mean (SD)**	**Min**	**Max**	**Mean dif**	**95% CI**	***p***
6 months	1.88 (0.53)	1.00	3.25	2.01 (0.66)	1.00	3.75	−0.135	(−0.311,0.041)	0.125
12 months	1.70 (0.44)	1.00	2.50	1.83 (0.53)	1.00	2.75	−0.130	(−0.312,0.051)	0.149
24 months	2.30 (0.66)	1.50	4.50	2.57 (0.84)	1.25	4.50	−0.261	(−0.473,−0.048)	0.018
36 months	2.42 (0.69)	1.00	4.00	2.59 (0.71)	1.00	3.75	−0.163	(−0.0378,0.052)	0.130
	**Group 3: Moderately Rough Neck**			**Group 4: Minimally Rough Neck**			**Paired Difference**		
	**Mean (SD)**	**Min**	**Max**	**Mean (SD)**	**Min**	**Max**	**Mean dif**	**95% CI**	***p***
6 months	2.93 (0.71)	1.75	5.25	2.88 (0.65)	1.75	4.75	0.050	(−0.142,0.242)	0.592
12 months	2.65 (0.72)	1.75	4.75	2.68 (0.68)	1.75	4.50	−0.033	(−0.221,0.154)	0.709
24 months	2.48 (0.58)	1.25	3.50	2.34 (0.60)	1.00	3,25	0.143	(−0.114,0.401)	0.252
36 months	2.10 (0.68)	1.25	4.25	2.01 (0.58)	1.00	3.00	0.088	(−0.259,0.434)	0.603

**Table 5:** Mean OHIP score and the mean difference for each of the seven domains before surgery and three months after connection with the calculated effect-size.

Domain	Mean-OHIP (SD)		Paired Difference			Effect-Size
	Before Surgery	Three Months after Connection	Mean Dif	95% CI	*p*	
functional limitation	2.30 (1.85)	1.14 (1.42)	1.16	(0.540,1.785)	0.001	0.63
physical pain	3.37 (2.06)	1.21 (1.55)	2.16	(1.440,2.886)	<0.001	1.04
psychological discomfort	2.52 (2.35)	0.65 (1.43)	1.87	(1.034,2.687)	<0.001	0.80
physical disability	2.12 (2.16)	0.44 (0.85)	1.68	(0.971,2.378)	<0.001	0.78
psychological disability	2.21 (1.91)	0.58 (0.93)	1.63	(0.930,2.326)	<0.001	0.85
social disability	1.67 (1.49)	0.16 (0.49)	1.51	(1.007,2.016)	<0.001	1.01
handicap	1.42 (1.48)	0.26 (0.66)	1.16	(0.683,1.642)	<0.001	0.78
